# Feasting on fish. Specialized function of pre-colonial pottery of the Cerritos mound builders of southern Brazil

**DOI:** 10.1371/journal.pone.0311192

**Published:** 2025-02-05

**Authors:** Marjolein Admiraal, André C. Colonese, Rafael Guedes Milheira, Alice Di Muro, Helen Marie Talbot, Alexandre Lucquin, Oliver E. Craig

**Affiliations:** 1 Department of Archaeology, BioArCh, University of York, York, United Kingdom; 2 Water and Environmental Research Center, University of Alaska Fairbanks, Fairbanks, Alaska, United States of America; 3 Department of Prehistory and Institute of Environmental Science and Technology (ICTA), Universitat Autònoma de Barcelona, Barcelona, Spain; 4 Department of Anthropology and Archaeology, Federal University of Pelotas, Pelotas, Brazil; University of Edinburgh, UNITED KINGDOM OF GREAT BRITAIN AND NORTHERN IRELAND

## Abstract

Some of the oldest coastal pottery in South America is found in the Pampas region of southern Brazil, Uruguay and Argentina. In the region’s extensive estuarine systems pre-colonial indigenous groups built earthen mounds, known as Cerritos, from ca. 4700 BP. The Cerritos have multifunctional purposes, and while pottery was widely used, its role in the economic or ritual life of the mound builders remains uncertain. Intriguingly, molecular and isotopic characterization of food residues from Cerritos ceramics shows that vessels were used for either cooking estuarine fish, or plant products. Microbial-derived lipids were predominantly associated with the latter, suggesting that plants were fermented, presumably to make alcoholic beverages. We suggest that dispersed communities were drawn to the mounds seasonally to exploit and celebrate the return of migrating fish. This finding is supported by the diversity of stable isotope values of human remains recovered from Cerritos and sheds new light on the lifeways of these pre-colonial groups.

## Introduction

**Fig 1:**
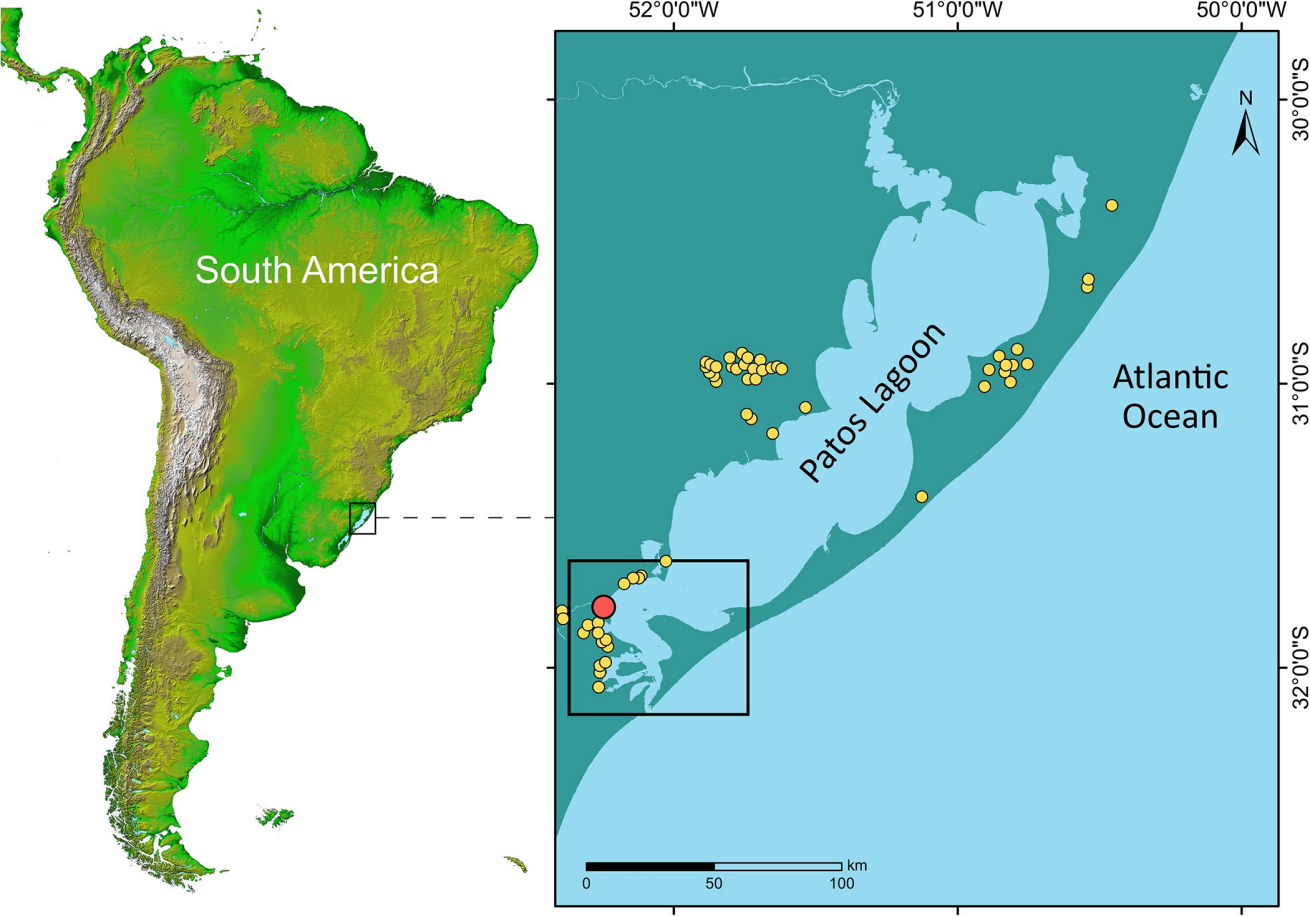
Map of South America, with the location of Cerritos sites in Patos Lagoon (yellow), study site highlighted in red. Map generated using ArcGIS 10.8 and Inkscape 1.1.2. We used publicly available data (CC BY 4.0) data from World Bank’s Data Catalog (https://datacatalog.worldbank.org/search/dataset/0038272/World-Bank-Official-Boundaries), Flanders Marine Institute (www.marineregions.org), and NASA/JPL-Caltech (adapted from https://www.jpl.nasa.gov/images/pia03388-south-america-shaded-relief-and-colored-height).

Coastal wetlands are among the most biologically productive ecosystems in the world, having supported longstanding Indigenous communities in pre-colonial South America for thousands of years [1–3]. Patos Lagoon in southern Brazil provides examples of complex human-coastal wetland and grasslands interaction during the Late Holocene [1, 4, 5], a human-environment connection that was all but wiped out with European colonization from the 16^th^ century. Today, our understanding of this lost connection relies solely on the valuable insights provided by the extensive regional archaeological record, and a few historical documents. Since ca. 3,500 years ago Patos Lagoon was occupied by the builders of distinctive earthen mounds locally known as *Cerritos de Indios* or *aterros* (Fig 1 and S1 Fig). The sites are the local expression of a larger cultural phenomenon that spread out across the Pampas biome of Uruguay, Argentina, and Brazil. They consist of organic-rich sediments of "dark earth" containing scattered archaeological remains such as pottery, lithics, botanicals, fauna, human bones, and teeth [4]. Cerritos date between 4700 to 200 cal BP and are found both isolated and in clusters. These clusters can incorporate up to a hundred mounds with circular or elliptical shapes. Mound diameters range from 30 to 60 m, and can be shallow, with only a few cm in height, or tall, up to seven meters [4, 6]. Their function remains elusive, variously interpreted as domestic, ceremonial sites, campsites, and cultivation plots [6–8]. Based on their location in areas that are subject to flooding, it has been suggested that Cerritos mounds provided a unique engineering adaptation to the local environment [9, 10]. There is evidence that these sites were the result of systematic seasonal occupations [4, 5], however, a few sites also contain stratigraphic layers, activity areas (fires, human burials, refuse, pits), and structural buildings, suggesting that these mounds were architectural planes for daily life and ritual purposes. Some sites were articulated to other structures such as micro-reliefs (mounds less than 30 cm in height), elongated platforms, borrow pits, tracks, pathways, and artificial lakes compounding archaeological complexes [4–9].

Human remains at these sites are scarce, and when found, they are often highly fragmented and disarticulated [11, 12]. This hampers the ability to obtain basic demographic information and insights into individual life histories, such as health and nutritional conditions. Stable carbon and nitrogen isotopic analysis of human bone from the earthen mounds of Uruguay [13], and the Paraná River delta in Argentina [14] suggests the Cerritos (Goya-Malabrigo tradition) had a mixed economy based on C_3_ plants, fish and terrestrial animals, while in the south Brazilian inland maize possibly contributed to the diet [15]. At Pontal da Barra, situated in the estuary of Patos Lagoon, southern Brazil, isotope analysis of 20 individuals showed the importance of aquatic resources (marine/estuary/freshwater) but also revealed great diversity in dietary regimes ranging from C_3_ plants/freshwater fish to marine and even a C_4_-based diet in one individual [5, 16]. The nature of this dietary variability remains uncertain, potentially stemming from exogenous factors such as long-distance social interactions, as well as endogenous processes including local social inequalities and food restrictions [5, 16].

Significantly, Cerritos in the Patos Lagoon area contains some of the earliest ceramic artifacts in southern Brazil, dating to ca. 3,000 cal BP [16–18]. Reconstructions and technological analysis of Cerritos pottery indicate predominantly plain, small bowl-like vessels with minor variations in surface treatments and made with local raw materials [10, 17, 19, 20]. These artifacts are contemporaneous to early ceramic vessels at the sites of Potrerillo de Santa Tereza and Cráneo Marcado in Uruguay [19, 21] where phytoliths of palm fruits (*Butia capitata*), cannanáceas (*Canaa* sp.) as well as maize (*Zea mays* L.), cucurbitas squash (*Cucurbita* sp.), and beans (*Phaseolus* sp.) were identified on ceramic surfaces [19]. Overall, the function of these small ceramic vessels was proposed to have been mainly utilitarian, for cooking, storage, or serving ware [10, 19]. Other interpretations suggest that Cerritos pottery was used to process fish either for immediate consumption or for extended preservation, this is in line with abundant fish remains at sites [4, 10]. However, ceramic artifacts found in funerary contexts suggest they also had a ceremonial value [11, 22].

Here we analyzed lipid residues preserved in 54 pottery sherds recently retrieved from two mostly contemporaneous Cerritos in Pontal da Barra, southern Brazil: PSG-02 (1859–1280 cal BP) and PSG-07 (2340–1214 cal BP, Fig 1) [16, 17]. The sites belong to an aggregation of 24 Cerritos, of which five were recently excavated and several multidisciplinary studies were applied to the archaeological remains [5, 11, 16, 17], making this an excellent case study to gain insights into the function of pottery in order to advance our understanding of the economy and overall role of the Cerritos in the Patos Lagoon region. In particular, we aim to assess the role of wetland resources, notably fish, in the longstanding debate on the economic foundation of Cerritos’ society.

## Materials and methods

Fifty-four pottery sherds from the site of Pontal da Barra in Patos Lagoon, southern Brazil were analyzed by organic residue analysis. Permits for organic residue analysis were obtained from the Instituto do Patrimônio Histórico e Artístico Nacional (IPHAN, protocol 01510.000608/2021-80, 01512.000594/2020-01, 01510.000612/2020-67, 01510.000422/2022-10). We compared our data to modern reference samples of fish (bone), obtained from Babitonga Bay in Brazil (S2 Table). Modern fish samples, previously analyzed by Fossile et al. [23] were commercially acquired in Joinville between 2018 and 2019 and registered in the Sistema Nacional de Gestão do Patrimônio Genético e do Conhecimento Tradicional Associado (SisGen, nº. RF54A7C and R22244B) following article 22 of Decree 8.772, of May 11, 2016. Information regarding the ethical, cultural, and scientific considerations specific to inclusivity in global research is included in the S1 Checklist.

### Organic residue analysis

Pottery (S1 Table) and modern fish bone (S2 Table) samples were extracted using acidified methanol and following existing protocols [24]. Briefly, after mechanically cleaning the pottery surface to avoid contamination (removal of ca. 1 mm of the surface), ceramic powder samples of ca. 1 g were obtained by drilling into the pottery sherd (ca. 2–5 mm). The modern fish bones (see S2 Table) were solvent-washed (3x 2 mL dichloromethane/methanol 2:1 v/v) before acidified methanol extraction of ca. 1 g of sample. Four mL of methanol was added to all samples (including a standard and blank per batch) and sonicated for 15 min after which 800 μL sulfuric acid was added. The samples were subsequently heated for 4 hours at 70 °C. Lipids were extracted with *n-*hexane (3 x 2 mL). Seven samples were also solvent extracted [25]. In short, the ceramic powder (ca. 1 g) was extracted using a dichloromethane/methanol 2:1 v/v mixture (3x 2 mL). Subsequently, the acid and solvent extracts were derivatized to their TMS esters using N, O-bis(trimethylsilyl) trifluoroacetamide (BSTFA). Samples were then analyzed by GC-flame ionization detection (GC-FID), GC-MS, and GC-C-IRMS (see S1 File for details). To facilitate comparison with our archaeological pottery data, modern δ^13^C isotope values of reference materials were corrected for the Suess effect, taking into account the year of death of the animal. Moreover, we estimated that the contribution of post-industrial carbon in the modern marine animal was 50% [26].

### Statistical analysis and mapping

Statistical analysis (Pearson R) was performed using R Studio (version 2022.07.2). The map (Fig 1) was generated using ArcGIS 10.8 (https://desktop.arcgis.com/en/) and Inkscape (https://inkscape.org/) on data publicly available (CC-BY NC 4.0) from i) GeoWeb Embrapa (https://mapas.cnpm.embrapa.br/apps/site_aquicultura/#/map), ii) the Flanders Marine Institute (www.marineregions.org), and iii) NASA/JPL-Caltech (https://www.jpl.nasa.gov/images/pia03388-south-america-shaded-relief-and-colored-height).

## Results

We analyzed the organic residues preserved in 54 pottery sherds from Cerritos PSG-02 (n = 30, of a total of 1220 sherds) and PSG-07 (n = 24, of a total of 864) in Pontal da Barra, southern Brazil. Sherds were selected from different layers and units throughout the sites aiming to avoid multiple sampling of the same vessel. Preservation was sufficient for interpretation (> 5 μg g^-1^) in all samples and lipid concentrations range from 7 to 988 μg g^-1^ (mean = 144 μg g^-1^) (S1 Table). Through the combined analysis of the molecular composition of the samples and the bimodal distribution of the stable carbon isotope values of palmitic (C_16:0_) and stearic acid (C_18:0_), we distinguished two main uses of the Cerritos pottery: 1) aquatic (marine/estuarine), reflected in high δ^13^C values of palmitic and stearic acids, high lipid concentrations, and the presence of aquatic biomarkers; and 2) plants, reflected in low lipid concentrations, low δ^13^C values of palmitic and stearic acids for C_3_ plants (with the exception of three samples potentially reflecting the processing of maize and portraying high δ^13^C_16:0_ and δ^13^C_18:0_ values), plant biomarkers, and ketones (K_29-33_). These groups are discussed in detail below.

### Marine products

**Fig 2:**
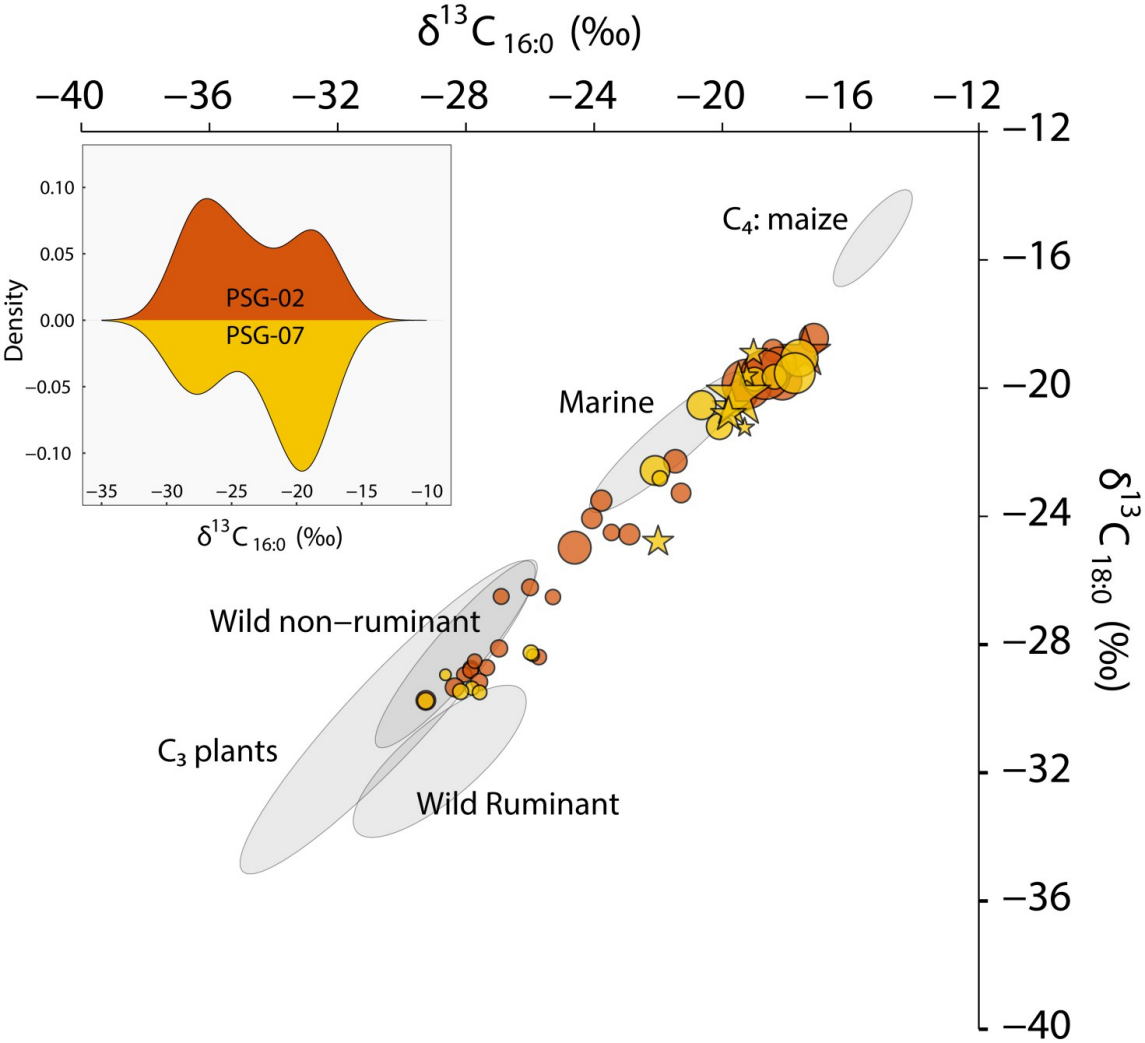
Carbon isotope values of palmitic (C_16:0_) and stearic (C_18:0_) acid of Cerritos ceramics from Pontal da Barra. Red = PSG-02; yellow = PSG-07, against 68% confidence ellipses based on modern reference materials (see S2 Table) and as a density plot illustrating the two use categories of Cerritos pottery. Star shapes refer to samples containing aquatic biomarkers in the form of APAAs with a C_20/18_ ratio of >0.06 [27], symbol size reflects lipid concentrations.

**Fig 3:**
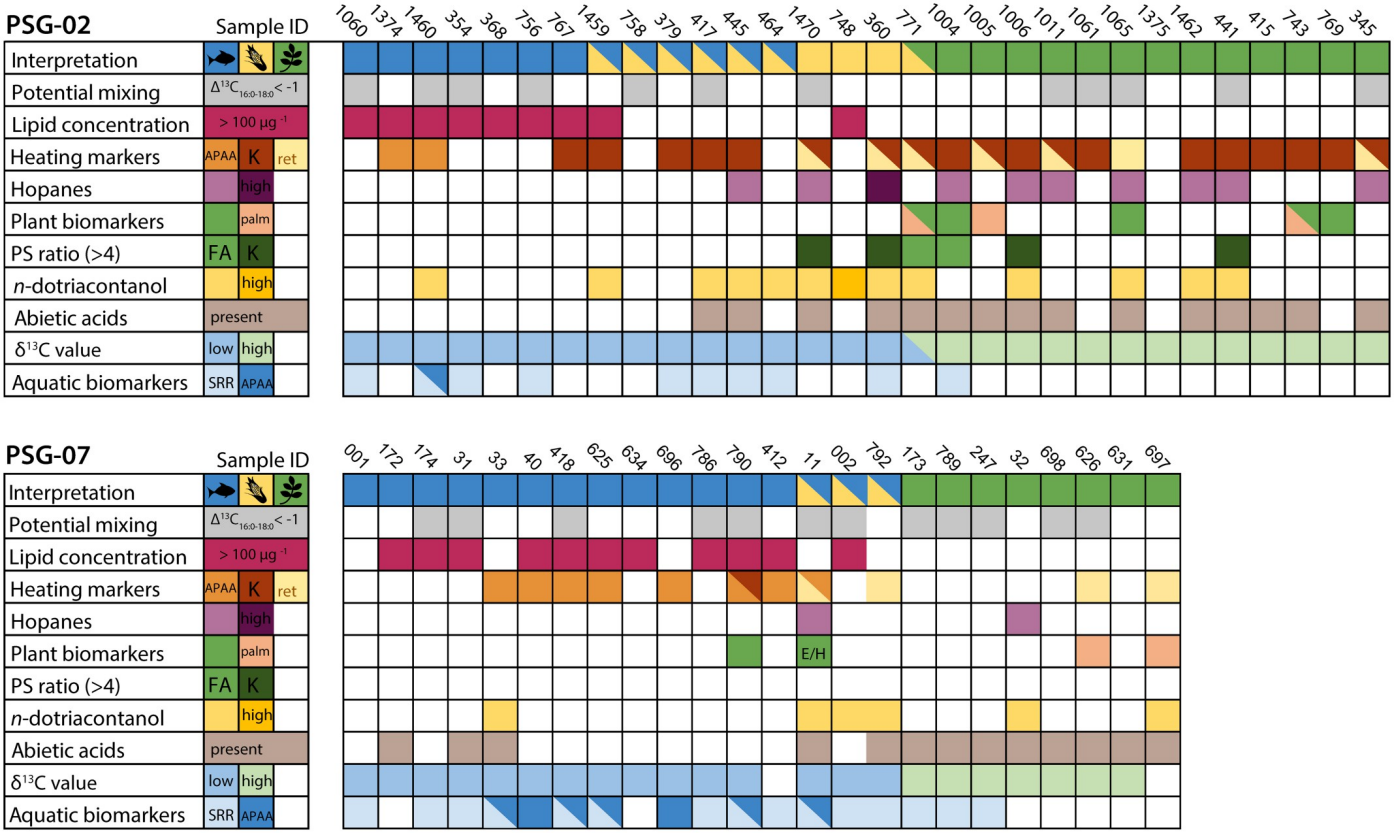
Overview of lipid residue biomarker and isotope results. Individual samples from PSG-02 and PSG-07 shown in columns, rows reflect interpretation of the samples as aquatic, maize and/or C_3_ plants; the potential of mixing in samples based on the offset of the isotopic values of C_16:0_ and C_18:0_ fatty acids [45]; lipid concentration (> 100 μg ^-1^); presence of heating markers: APAAs, ketones, and retene [28]; hopanes [35]; plant biomarkers (sterols, terpenes, APAA E/H ratio [27], and high % C_12:0_ reflecting the potential presence of palm products [30]; P/S ratios over 4 from fatty acids and reconstituted P/S ratios from ketones [37]; the presence of *n-*dotriacontanol [40]; abietic acid and derivatives [34]; high and low δ^13^C values of fatty acids and ketones (the latter only in sample 771); and the presence of aquatic biomarkers (APAAs C_20/18_ ratio > 0.06, and SRR isomer >75.5% [31]).

Eight of 54 samples (14.8%) had aquatic biomarkers in the form of ω-(o-alkylphenyl) alkanoic acids (APAAs) with a C_20/18_ ratio of >0.06 [27], or dihydroxy acids (DHAs). These compounds are formed from mono-, di-, and tri-unsaturated fatty acids present in aquatic oils. Specifically, APAAs will form when these compounds are subjected to prolonged heating [27–30]. Interestingly, nearly all of the samples with APAAs occur in Cerritos PSG-07, indicating that aquatic resources were extensively heat processed in the ceramics from this mound. A total of 25 samples (46%), equally distributed across the two mounds, presented further evidence for the presence of aquatic resources in these pots, reflected in the abundance of the SRR diastereomer of phytanic acid (SRR% >75.5) and the presence of TMTD [31]. The molecular evidence is further supported by high δ^13^C values of C_16:0_ and C_18:0_, with values plotting in the range of modern reference fats of marine and estuarine species. These samples (n = 30) presented high lipid concentrations (mean = 236 μg g^-1^, see Fig 2). Indeed, we observe a strong correlation between lipid concentrations and δ^13^C values (Pearson r = 0.576, df = 49, t = 4936, p = <0.05) in our data (Figs 2 and 3, S1 Table).

### C_3_ plants

Samples with low δ^13^C values of C_16:0_ and C_18:0_ along with low lipid concentrations (n = 21, mean = 17 μg g^-1^) are consistent with the processing of C_3_ plants compared to other terrestrial products. Plant biomarkers (Stigmasterol, β-Sitosterol, and β-Amyrin) were present in six samples, and five samples yielded high proportions of lauric acid (C_12:0_) relative to the other saturated fatty acids (C_14:0–18:0_), possibly reflecting the processing of palm kernel oil in these vessels [32, 33]. Abietic acid derivatives (dehydroabietic acid, 7-oxo-dehydroabietic acid and retene) were present in 28 samples (5 in trace amounts), which may indicate the use of coniferous resins for coating or sealing pottery [34, 35], while these compounds may also enter the ceramic matrix through firing or cooking using wood as fuel [36]. Eighteen samples contained mid-chain ketones K_29-33_, of which 16 had reconstituted P/S ratios of 2.1–5.4 presenting good evidence for the plant origin of these heating markers as ketones are unlikely to be affected by diagenesis [37]. Several samples (n = 7) had large abundances of *n*-alkanes with average chain lengths (ACL) ranging from 26.6 to 31.2, a carbon preference index (CPI) of 0.98 to 1.03 indicates that these n-alkanes are likely derived from a petroleum source and are therefore likely the result of contamination [38–40].

### Maize

Long-chain *n*-alkanols includin*g n*-dotriacontanol (C_32_), were observed in 19 samples. Of these, 13 had high δ^13^C values of palmitic and stearic acid (Figs 2 and 3), and 9 of those lacked aquatic biomarkers and had relatively low lipid concentrations (mean = 93 μg g^-1^). Two of these samples had mid-chain ketones with reconstituted P/S ratios (4.1 and 6.8) reflective of plants. While the abundance of the *n*-dotriacontanol was not sufficient to directly measure its isotopic value, considering the other evidence, we cautiously interpret these samples as potentially representing the processing of maize [41–43]. In one sample (PSG-02-771) we were able to measure the δ^13^C values of ketones K_31_ and K_33_, which confirmed a C_4_ plant origin with high δ^13^C values (ca. -22‰). Interestingly, the δ^13^C values of the free fatty acids in this sample were comparable to those of C_3_ plants (ca. -28‰, see S1 Table). Other evidence also points to the processing of plants in this sample (e.g., low lipid concentration of 23 μg g^-1^, high lauric acid %, and a high P/S ratio of 6.2) and might reflect the mixing of C_3_ and C_4_ plant products. Interestingly, although oleic acid (C_18:1_) is generally abundant in plants (e.g. maize, palm kernel and pulp, nuts, tubers, beans, squash), it is absent in most Cerritos samples (n = 52), perhaps due to degradation. Likewise, diacid C_9:0_, a degradation product of oleic acid, is also absent in these samples.

### Heating markers

The majority of samples (n = 33, 61%) have evidence of thermally altered lipids, providing unequivocal evidence for the heat transformation of products during cooking. These include mid-chain ketones, formed from the heating of animal and/or plant oils [44, 45], APAAs as described [27, 28]; and retene, formed from heating coniferous resins [34]. Interestingly, 19 of the 20 samples that yielded mid-chain ketones were from mound PSG-02 as are 7 of the 11 samples containing retene. In contrast, 8 of 10 samples with APAAs were from PSG-07, which points to different processing/cooking methods between the two locations. As ketones are formed at higher temperatures than APAAs [27, 28, 43, 44] this may reflect differences in the heat applied to the vessels. While the relationship between the formation of heating markers and the culinary processes/cooking techniques is not yet fully understood, and further experimental work is needed, we observe interesting patterns in the distribution of heating markers in the Pontal da Barra pottery assemblage. Only one sample (PSG7-790) yielded both ketones and APAAs. APAAs are mainly limited to marine samples (9 out of 10), while ketones and retene seem to occur predominantly in samples interpreted as C_3_ plants or maize, suggesting that these products were subjected to different culinary processes.

## Discussion

### Specialized use of pottery by the Cerritos mound builders of Patos Lagoon

The organic residue analysis of Cerritos pottery from Patos Lagoon revealed two main products: marine resources and plants, the latter including maize. Interestingly, there is only limited evidence for mixing of these two sources. Based on our results, we hypothesize that pottery at Pontal da Barra was deliberately produced for the processing and consumption of these specific resources in the context of seasonal harvesting of fish and feasting, much in the way that the Guarani produced pottery specifically for feasting events [52]. The distribution of heat altered lipids shows that specific modes of cooking were deployed and that these had a spatial dimension with differences between mounds. The use of utilitarian cooking pots reflecting the co-, or sequential, processing of different plant and animal products, as extensively documented at ceramic Atlantic forest sites [45], is less well supported here. Furthermore, terrestrial animal fats were not apparent despite the presence of ruminant animals and rodents in the faunal assemblage [5]. So why were marine fish only found in a selection of pots analyzed, and why were these rarely found mixed with wild or cultivated plants?

### Feasting on fish

One explanation is that the usage patterns are the results of seasonal or episodic use of pottery when fish were in high abundance. The whitemouth croaker (*Micropogonias furnieri*) is a coastal/estuarine teleost fish that often dominates the faunal assemblage at Cerritos mounds. These were likely caught together with marine catfish (*Genidens* sp.) during the summer spawning season, when they are at particularly high abundance [17, 46, 47]. Although no seasonality data is available at the Cerritos sites, studies of whitemouth croaker otoliths from mid- to late-Holocene sites in the northern Río Negro in Argentina consistently show that they were captured between November and January [48]. We suggest that Cerritos pottery was, at least in part, produced to maximize the return from these seasonally abundant fish, through processing for long-term preservation. Investment in pottery production in anticipation of seasonal aquatic resources is thought to be a major driver for its uptake in north-eastern North America, where it is proposed that dispersed groups aggregated at certain times of the year to participate in collective mass harvesting of fish and undertake other social activities, such as funerals and marriages [49], a practice also known from other areas [50].

A similar socio-economic mechanism might explain the appearance of pottery at the Cerritos mounds. Hayden emphasizes the role of pottery, over other organic containers, as a prestige technology that would have been overtly displayed during competitive feasts [51]. Although seasonal occupation of the Cerritos was proposed decades ago [10] and dismissed more recently in favor of a more permanent settlement [5, 17], we argue that some aspects of the activities at these mounds, particularly associated with harvesting, fishing, and feasting, may indeed have had a seasonal component. This hypothesis is supported by the stable isotope analysis of human remains recovered from the Patos Lagoon Cerritos, not reflecting the consumption on site, but showing that individuals had distinct dietary histories, with a substantial number of predominantly terrestrial consumers, indicative of a broad social catchment [5]. It is likely that individuals from outside the coastal community either visited the site, or else their remains were brought to the site for secondary burial. Evidence of funerary activity fits well with seasonal feasting dictated by the annual return of spawning fish.

Seasonal aggregation for intensive aquatic resource exploitation invites questions regarding the role of pottery in the processing, storage, and consumption of fish products. Heating markers were found in approximately half the pots with fish (11 of 21), which might be the result of the extraction of fish ‘meal’ (or ‘flour’) or the rendering of oil, but equally as fish stews, likely for more immediate consumption. Historic indigenous groups on the coast of Brazil were observed to have used pottery for these purposes [52]. The absence of heating markers in pots containing marine oils is intriguing since they are readily formed when heated [27]. It is possible that fish were fermented rather than cooked to facilitate their long(er)-term storage. Although pottery is an abundant find at Pontal da Barra, regrettably, it consists mainly of small, fragmented sherds of plain ceramics, preventing reconstruction of vessel shape and volume to investigate specialized use in more detail. Traits such as fabric (mainly coarse inclusions of quartz and feldspar) or surface treatments (mainly smooth surfaces) also did not allow for discrimination between samples. Sherd thickness shows no correlation to our results (isotopic values: Pearson r = -0.0098, df = 31, p = 0.958; lipid concentration: Pearson r = -0.18, df = 31, p = 0.321; interpretation categories: Pearson r = 0.15, df = 30, p = 0.422; and aquatic biomarkers: Pearson r = -0.33, df = 32, p = 0.052).

### Fermentation of plants?

A range of C_3_ and C_4_ plants such as squash, beans, tubers, palm, peanuts, and maize, were widely exploited by the Cerritos mound builders, as shown by archaeobotanical research [4, 6, 13, 19, 53, 54]. We were able to corroborate evidence for maize in a limited number of vessels (9 potentially, 4 more confidently: see Fig 3). Distinguishing different C_3_ plants is more challenging, and we expect that starch rich tubers, legumes, and squashes leave minimal lipid residues due to their very low lipid content. High abundances of lauric acid in five samples are consistent with palm kernel oil, this is supported by the presence of lithic artifacts at the Cerritos sites known as *quebra-coquinhos*, used for breaking open the butiá nuts of palm trees [21].

The presence of heating markers associated with plant residues in PSG-002 and their absence in PSG-007 is intriguing and suggests that plants were treated differently spatially at Pontal da Barra. Cooking or roasting was often an important step in the preparation of fermented beverages among Brazilian indigenous groups, especially for the fermentation of cassava and maize based beverages [55, 56]. Heating is, however, not a requirement for fermentation. The lack of heating markers in mound PSG-007 may reflect the use of a different fermentation technique or a different use of the pottery altogether, for example for storage or as serving ware. In indigenous fermentation practices bowls are known to have been used to collect chewed plant materials, the saliva containing the Diastase enzyme which aids in the fermentation process as it allows the conversion of starches to sugars [55, 56]. Hopanes (C_30-32_), with a base peak of *m/z* 191, previously interpreted as markers of fermentation [35, 57], were found in pots with both C_3_ plant and maize residues (see Fig 3; S1 Table). These compounds are the degradation products of bacteriohopanoids derived from a wide range of bacteria, and as such, are ubiquitous in soils as well as modern and ancient sediments and may also derive from petroleum products [58]. While their occurrence does not correspond to pots with or without heating markers, it is noteworthy that these compounds are rarely present in vessels containing aquatic derived lipids (n = 1). Similarly, all samples with hopanes also contain the diterpenoid, abietic acid, derived from conifer resin, and the latter is dominant in samples with plant residues (Fig 3). It might be that conifer resins were used as liners to seal vessels used to hold or ferment liquids [35]. Although the evidence for fermentation is not conclusive, the correspondence between plant biomarkers, conifer resin and bacterial derived lipids is intriguing.

There is ample historical evidence of fermentation by Indigenous groups of the South American lowlands. The sap of the Acuri or Buriti palm is documented to have been fermented by the *Guató* from the Pantanal biome, while the *Coroados* from southern Brazil used to extract and ferment tree sap from the trunk of the palm tree [59, 60]. Guarani groups also made a fermented beverage (*Mapuitã Rykueof*) of palm (*Syagrus romanzoffiana*) named Pindó [61, 62]. Today, the *mel do butiá* (honey of the palm fruit) is used as a syrup for respiratory diseases and as an alcoholic drink, linking recent history to the past traditional indigenous use of this plant [63]. Among the Guarani, fermented beverages are thought to have played an important role in rituals [52] and were essential for forging political relationships between groups, allowing them to mobilize, control, and spread manpower [64]. They are suggested to have played a major role in the spread and adoption of pottery in South America [65]. Given the other evidence, the potential role of fermented beverages in feasting during periods of seasonal aggregation in Cerritos’ society is compelling.

## Conclusion

The specialized pottery usage patterns described here for the Cerritos mound builders stand out compared to previous studies of pottery by lowland coastal South American groups. While the contemporary Taquara-Itararé pottery (ca. 1200–900 cal BP) found to the north of Patos Lagoon in the coastal areas of Santa Catarina island, Babitonga Bay and Laguna was used to process a range of marine/estuary resources, terrestrial animals and C_3_ plants [26, 33, 45, 66], frequent mixing of these resources shows a primarily utilitarian function. Similarly, pottery use by the Guarani, that superseded the Cerritos of the Pampas and the Taquara-Itararé on the Atlantic forest coast, shows extensive mixing of maize with terrestrial plants and animals. Guarani pottery, specifically from Patos Lagoon, is an interesting exception in that it was not used for processing maize or marine resources, but instead was used to process C_3_ plants [45]. The reason for the absence of maize in Guarani pottery from this region remains unclear, but likely sustainable cultivation was not possible in the saline environment [67]. While the presence of maize in Cerritos pottery, potentially as a fermented beverage, from the same region may imply that it was brought to the site specifically for ritual feasting. We suggest that these sites, at least in part, functioned as prominent monuments in a frequently flooded landscape conducive to seasonal mass capture of fish, and that social aggregation and ritual feasting were major activities. Pottery, perhaps as a prestige technology, was deeply integrated in this complex socio-economic system. Therefore, this study contributes to a wider understanding of the significance of coastal wetlands to pre-colonial indigenous societies in South America.

## Supporting information

S1 Fig Aerial image showing the flooding location of the Cerritos from Pelotas, southern Brazil: A) Pavão 01 site, located by the São Gonçalo channel; B) the Pontal da Barra sites between the São Gonçalo channel and the Patos Lagoon (Images by Rafael Milheira). (JPG)

S1 File Supporting information. Supporting information Methods and Materials (instrumentation).
(DOCX)

S1 Table ORA data Cerritos. Biomolecular and stable isotope results from Cerritos pottery analysed in this study.
(XLSX)

S2 Table Compound specific isotope reference data. Compound specific isotope values of fatty acids from reference materials using in this study.
(XLSX)

S1 Checklist Questionnaire on inclusivity in global research. (DOCX)
