# Supplementary figures and images for: Feasting on fish. Specialized function of pre-colonial pottery of the Cerritos mound builders of southern Brazil
Source: PLoS One. 2025 Feb 5;20(2):e0311192. doi: 10.1371/journal.pone.0311192 (PMC11798503; doi:10.1371/journal.pone.0311192)

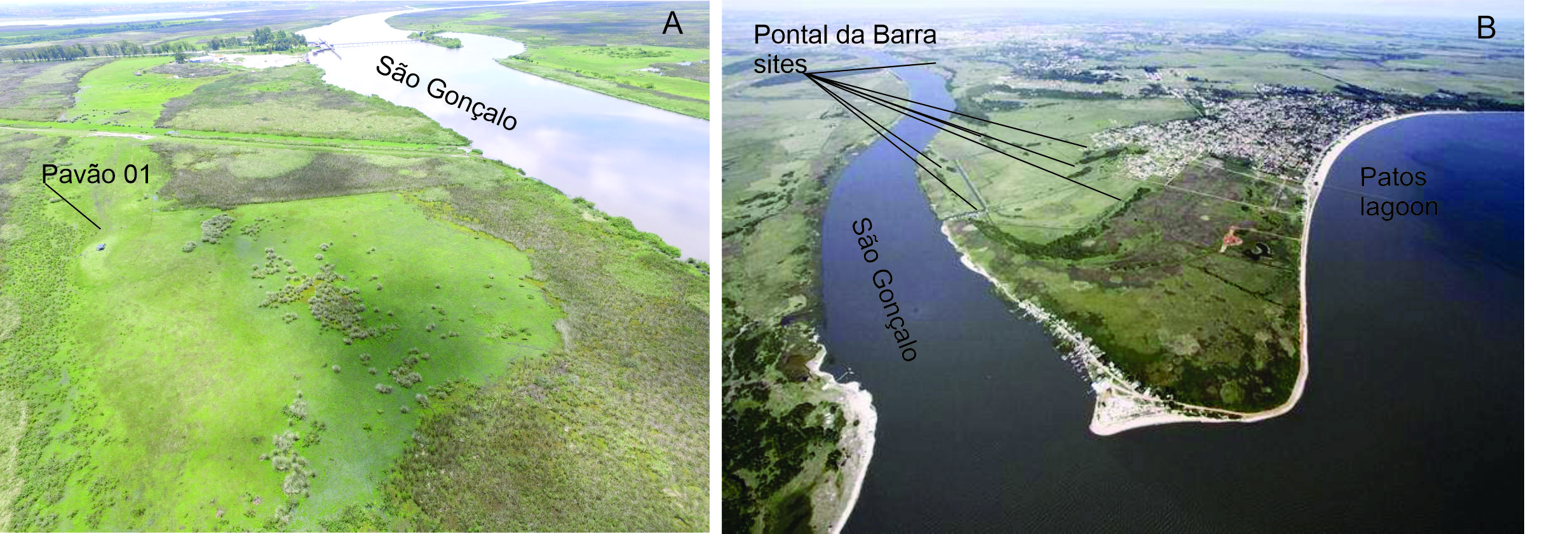

Supplement: S1 Fig — (JPG) [file pone.0311192.s001.jpg]
