# Supplementary material for: Feasting on fish. Specialized function of pre-colonial pottery of the Cerritos mound builders of southern Brazil
Source: PLoS One. 2025 Feb 5;20(2):e0311192. doi: 10.1371/journal.pone.0311192 (PMC11798503; doi:10.1371/journal.pone.0311192)
Supplement: S1 File — Supporting information Methods and Materials (instrumentation). (DOCX) [file pone.0311192.s002.docx]

Supplementary Information for

**Feasting on fish. Specialized function of pre-colonial pottery of the Cerritos mound builders of Southern Brazil.**

Admiraal *et al.*

*Corresponding author. Email: marjoleinadmiraal@gmail.com

**This PDF file includes:**

Supplementary Text

**Other Supplementary Materials for this manuscript include the following:**

S1 Fig, S1 Table, S2 Table, S1 Checklist

**Instrumentation**

Gas Chromatography - Flame Ionization Detector (GC-FID)

Screening and quantification of all samples was done by GC-FID (Agilent 7890A gas chromatograph from Agilent Technologies, Cheadle, Cheshire, UK). One µL of sample suspended in hexane was injected into the GC, the splitless injector was set to 300°C. The column used was a 100% Dimethylpolysiloxane DB-1 (15 m x 320 µm x 0.1 µm; J&W Scientific, Folsom, CA, USA). Helium was used as a carrier gas set at a constant pressure of 16.6 psi. Flow rate was set to 2 mL min^-1^ (constant). The temperature program was as follows: 100°C for 2 min, increasing with 20°C min^-1^ until 325°C. This temperature was maintained for 3 min. Total run time: 16.25 min.

GC - Mass Spectrometry (GC-MS)

Several instruments were used for the GC-MS analysis of the samples: 1) Agilent 7890A series GC connected to Agilent 5975C Inert XL mass-selective detector with a quadrupole mass analyzer (Agilent Technologies, Cheadle, Cheshire, UK); 2) Trace Ultra 1310 GC, coupled with an ISQ MS (Thermo Fisher, Bremen, Germany); and 3) Perkin Elmer CLARUS 690 SQ 8T GC-MS. The GC configuration and temperature programs were the same and reproducibility was tested by measuring lipid standards added to each run.

Scanning was performed using a DB-5ms (5%-phenyl)-methylpolysiloxane column (30 m × 0.250 µm × 0.25 µm; J&W Scientific, Folsom, CA, USA) and directly inserted into the MS ion source. The injector (splitless mode) kept at 300°C. Helium was used as the carrier gas (flow rate: 3 mL/min). Scanning to obtain spectra occurred between *m/z* 50 and 800 (MS ionization energy: 70 eV). The temperature program was as follows: 50°C (2 min) then increasing by 10°C min^-1^. At 325°C the temperature was held for 15 min. To quantify aquatic biomarkers (ω-(o-alkylphenyl) alkanoic acids and isoprenoid acids) all acid extracted samples were analyzed with instrument 1 equipped with a DB-23 (50%-cyanopropyl)-methylpolysiloxane column (PN 122-2362; 60 m × 0.250 µm × 0.25 µm; J&W Scientific) in SIM mode (single ion monitoring) (see 1). The injector (splitless mode) was set at 300°C. The temperature program was as follows: 50°C (2 min), then increasing by 10°C min^-1^ to 100°C. Then increasing with 4°C min^-1^ to 140°C, then with 0.5°C min^-1^ to 160°C, and then with 20°C min^-1^ to 250°C, where it was maintained for 10 min. Spectra for the several compounds were acquired by scanning ions specific for the different biomarkers: 4,8,12-trimethyltridecanoic acid (TMTD: *m/z* 74, 87, 213, 270), pristanic acid (*m/z* 74, 88, 101, 312), phytanic acid (*m/z* 74, 101, 171, 326) and ω-(o-alkylphenyl) alkanoic acids (APAA) of carbon length C^16^ to C^22^ (*m/z* 74, 105, 262, 290, 318, 346). Helium was used as the carrier gas with a constant flow rate of 1.5 mL/min. MassHunter software (version B.07.01, Agilent Technologies was used for the quantification of compounds and integration of specific ions depending on the compound: saturated and branched fatty acids and TMTD (*m/z* 87), pristanic acid (*m/z* 88) phytanic acid (*m/z* 101), APAA C^18:0^ (*m/z* 290), APAA C^20:0^ (*m/z* 318), and *n-*alkanols (*m/z* 75).

GC-combustion-Isotope Ratio MS (GC-c-IRMS)

We analyzed 194 samples by GC-c-IRMS following existing protocol (2) in order to obtain the stable isotope values of palmitic (C_16:0_), stearic (C_18:0_) and oleic acid (C_18:1_), using an Isoprime 100 (Isoprime, Cheadle, UK) connected to a Agilent 7890B Series GC (Agilent Technologies) with a Isoprime GC5 interface (Isoprime). We used ultra-purity grade Helium as a carrier gas (flow rate of 2 mL/min). One µL of sample (diluted in hexane) was injected into a DB-5MS ultra-inert fused-silica column at 300°C (PN 122-5562UI; 60 m x 250 µm x 0.25 µm; J&W Scientific). The temperature program was as follows: 50°C (0.5 min), increasing by 25°C min^-1^ to 175°C, then increasing with 8°C min^-1^ to 325°C where it was maintained for 20 min.

Electron impact in the MS allowed for ionization of eluted products. Ion intensities (*m/z* 44, 45, 46) were recorded and the ^13^C/^12^C ratio of each peak in the sample was computed automatically. Data analysis was done using the software IonVantage and LyticOS (Isoprime, Cheadle, UK). Results, relative to a international standard (VPDB) were expressed in per mill (‰). The accuracy of the instrument was monitored based on δ^13^C values (expected/measured) of *n*-alkanes and *n*-alkanoic acid ester international standards (Indiana A7 and F8-3). Measured mean values of Indiana standard F8-3 were -29.87 ± 0.15‰ for the methyl ester of C_16:0_ (reported mean value vs. V-PDB -29.90 ± 0.03‰), and 23.22 ± 0.18‰ for the methyl ester of C_18:0_ (reported mean value vs. VPDB -23.24 ± 0.01‰). All samples were measured in replicate (mean of S.D. 0.13‰ for C_16:0_, and 0.12‰ for C_18:0_). After analysis values were corrected by a mass balance formula to account for the methylation of the carboxyl group occurring during acidified-methanol extraction. An in-house standard mixture of C_16:0_ and C_18:0_ fatty acids with known isotopic composition was processed with each batch and was used for further. Isotope values of modern fish references (Dataset S2) were corrected for the Suess Effect based on the year of death (3).

**References**

1. L. J. Cramp, R. P. Evershed, Reconstructing aquatic resource exploitation in human prehistory using lipid biomarkers and stable isotopes. Treatise on Geochemistry 14, 319–339 (2014).
2. O. E. Craig, et al., Distinguishing wild ruminant lipids by gas chromatography/combustion/isotope ratio mass spectrometry. Rapid Commun. Mass Spectrom. 26, 2359–2364 (2012).
3. H. Hellevang, P. Aagaard, Constraints on natural global atmospheric CO^2^ fluxes from 1860 to 2010 using a simplified explicit forward model. Sci. Rep. 5, 17352 (2015).
